# The Temperature Dependent Proteomic Analysis of *Thermotoga maritima*

**DOI:** 10.1371/journal.pone.0046463

**Published:** 2012-10-05

**Authors:** Zhuowei Wang, Wei Tong, Quanhui Wang, Xue Bai, Zhen Chen, Jingjing Zhao, Ningzhi Xu, Siqi Liu

**Affiliations:** 1 Beijing Institute of Genomics, Chinese Academy of Sciences, Beijing, People's Republic of China; 2 The Department of Medicine, University of Louisville, Louisville, Kentucky, United States of America; Lawrence Berkeley National Laboratory, United States of America

## Abstract

*Thermotoga maritima* (*T. maritima*) is a typical thermophile, and its proteome response to environmental temperature changes has yet to be explored. This study aims to uncover the temperature-dependent proteins of *T. maritima* using comparative proteomic approach. *T. maritima* was cultured under four temperatures, 60°C, 70°C, 80°C and 90°C, and the bacterial proteins were extracted and electrophoresed in two-dimensional mode. After analysis of gel images, a total of 224 spots, either cytoplasm or membrane, were defined as temperature-dependent. Of these spots, 75 unique bacterial proteins were identified using MALDI TOF/TOF MS. As is well known, the chaperone proteins such as heat shock protein 60 and elongation factor Tu, were up-regulated in abundance due to increased temperature. However, several temperature-dependent proteins of *T. maritima* responded very differently when compared to responses of the thermophile *T. tengcongensis*. Intriguingly, a number of proteins involved in central carbohydrate metabolism were significantly up-regulated at higher temperature. Their corresponding mRNA levels were elevated accordingly. The increase in abundance of several key enzymes indicates that a number of central carbohydrate metabolism pathways of *T. maritima* are activated at higher temperatures.

## Introduction

Deciphering the thermophilic regulation of thermophiles at a high temperature environment is of particular interest in bacteriological research [1]. Generally, there are three factors considered to be involved in temperature adaptation: 1) changes in the expression level in response to temperature changes; 2) changes in the regulation of translation and post-translational modifications and 3) differences in the stability of proteins at different temperatures, either intrinsic or extrinsic. Currently, few studies have reported the relationship between gene expression and thermal stress [2]–[4]. With an analysis of 72 fully sequenced genomes, Paz *et al.* postulated that mixed adenine-guanine and polyadenine tracts within mRNAs of thermophiles mainly contributed to thermoadaptation [2]. After evaluating the substitutional asymmetries of the proteins from mesophiles and thermophiles, McDonald *et al.* found an inconsistent pattern of asymmetry for amino acids and suggested that the bioenergetic cost of amino acids or G+C contents could play an important role in temperature adaptation [6], [7]. Nevertheless, these genomic or transcriptional data have not provided much insight into thermophilic regulation of thermophiles at the protein level, which represent the functional modules in cell. A proteomics survey in response to culture temperature change may provide fundamental insights into understanding the comprehensive protein responses of thermophiles. We thus propose that protein profiling is an effective approach, which can bridge the thermophilic phenomenon and the functional molecules.

*T. maritima*, one of the lineages of hyperthermophilic heterotrophs, can grow anaerobically at temperatures above 90°C by obtaining its metabolic energy from substrate-level phosphorylation rather than respiration [3], [4]. It possesses some high temperature resistant enzymes, such as cellulases and hemicellulases, which could benefit the conversion of renewable biomass to fuels and chemicals, while avoiding environmental contamination. It is also an ideal model to study temperature-dependent gene expression, as it can survive within a wide temperature range, typically from 55 to 95°C. The genome of *T. maritima* was completely sequenced in 1999 [5]. According to the annotation of the *T. maritima* genome and functional studies of individual *T. maritima* proteins [6], [7], this bacterium contains many proteins that share high amino acid sequence homology with orthologs of mesophilic bacteria. Hence, a question naturally emerges; how are proteins with high homology in amino acid sequence between thermophiles and mesophiles able to perform under completely different thermal tolerances? An initial analysis of the proteome of *T. maritima* involved structural determination [8]. A total of 1376 genes from this bacterium were cloned into *E. coli* expression vectors, and the recombinant proteins were crystallized and analyzed in a high-throughput structural determination pipeline. Pysz *et al.* [9] analyzed *T. maritima* mRNAs that respond to heat shock during a temperature shift from 80 to 90°C, using a targeted cDNA microarray with 409 open reading frames. Although some of the regulatory responses were found at the mRNA level, there is still lack of reports on large-scale protein expression responses of this bacterium during environmental temperature changes.

In this study, for the first time, we report a proteomic survey of *T. maritima* at four temperatures, 60, 70, 80, and 90°C. The cytoplasmic and membrane proteins were carefully extracted from the bacterial cells, and then resolved by two-dimensional electrophoresis (2DE). Using image analysis and MALDI TOF/TOF MS, the temperature-dependent proteins were identified, including 41 unique proteins in cytoplasm and 37 in the membrane fraction. The identified proteins were functionally categorized, and this process suggested that a number of them were the enzymes participating in central carbohydrate metabolism. Combining data gained from real-time PCR and Western blot analyses, we come to the conclusion that up-regulation of protein abundances in central carbohydrate metabolism is likely a causal factor for thermoadaption in *T. maritima*.

## Materials and Methods

### 1 Chemicals

All of the chemicals employed for electrophoresis, such as IPG strips and ampholytes, were purchased from Amersham Biosciences (Uppsala, Sweden). α-Cyano-4-hydroxycinnamic acid (CHCA) was obtained from Bruker Daltonics (Bremen, Germany). Modified trypsin (sequencing grade) was from Promega (Madison, WI, USA). PVDF films were purchased from Millipore Corporation (Billerica, MA, USA). All the other reagents were of analytical reagent grade or the highest purity available.

### 2 Bacterial culture conditions

*T. maritima* strain MSB8 (JCM10099), a gift from Institute of Microbiology, Chinese Academy of Sciences, was activated and cultured at 80°C in modified medium containing (per liter of distilled water) 10.0 g starch, 5.0 g yeast extract, 10.0 g tryptone, 27.0 g NaCl, 0.5 g KH_2_PO_4_, 0.5 g K_2_HPO_4_, 0.5 g MgCl_2_·6H_2_O, 0.33 g KCl, 1.0 g NH_4_Cl, 0.08 g CaCl_2_·2H_2_O, 0.5 g L-cysteine, 0.5 g Na_2_S·9H_2_O, 1.0 mg resazurin and 15.0 ml of trace minerals solution containing (per liter distilled water) 1.5 g nitrilotriacetic acid, 3.0 g MgSO_4_·7H_2_O, 0.5 g MnSO_4_, 1.0 g NaCl, 0.1 g FeSO_4_·7H_2_O, 0.1 g CoSO_4_·7H_2_0, 0.1 g ZnSO_4_·7H_2_O, 0.01 g CuSO_4_·5H_2_O, 0.01 g AlK(SO_4_)_2_, 0.01 g H_3_BO_3_ and 0.01 g Na_2_MoO_4_·2H_2_O. The medium was boiled under a stream of oxygen-free N_2_ gas and cooled to 80°C. Two milliliters of the activated bacteria were inoculated to 100 ml of medium and then cultured at 60, 70, 80 or 90°C. To monitor the growth rates, the cultured bacteria were withdrawn at time intervals, every 2 hours, and their O.D._600 nm_ was measured. The growth curves of the bacteria were generated by fitting the data to the regression equation A = A_0_+a/(1+Exp(T_1/2_−*t*)/b), in which A is the O.D. value at 600 nm, *t* is the culture time, A_0_ is the O.D._600 nm_ at lag phase and T_1/2_ is the time when the cultures reach the middle of log phase. The “a” constant is the maximum change in the O.D._600 nm_ between A_max_ and A_0_, while “b” is the time between t_lag end_ and t_log end_. When the bacteria reached T_1/2_, the cells were considered at optimal conditions; the bacteria were harvested by centrifuge at 6,000 g for 15 min at 4°C followed by a wash with ultrapure water. The bacterial cultures at each temperature were prepared in triplicate.

### 3 Protein extraction

The washed cell pellets were disrupted by supersonication (750 W at 29% intensity) for 10 min in ultrapure water containing PMSF (1 µmol/ml), PVPP (10 µg/ml), RNase (10 µg/ml) and DNase (10 µg/ml). The cell debris was removed by centrifugation at 20,000 g for 30 min at 4°C, and the supernatant was subsequently subjected to ultracentrifugation at 170,000 g (SW32.1Ti, Beckman) for 2 h at 4°C. The pellet and supernatant generated from the ultracentrifugation were used as the membrane and cytoplasm fractions, respectively. The proteins in the cytoplasm fractions were precipitated in pre-cooled 10% TCA in acetone containing 50 mM DTT and placed on ice at −20°C for 3 h, followed by centrifugation at 35,000 g for 20 min. The precipitates were washed with pre-cooled acetone containing 10 mM DTT, 1 mM PMSF and 2 mM EDTA and dried in a Speedvac. The dried pellets were finally suspended in lysis buffer A containing 9 M urea, 4% CHAPS, 50 mM DTT, 0.5% ampholyte (pH 3.0–10.0), 1 mM PMSF, 2 mM EDTA and 20 mM Tris-HCl at pH 9.0, followed by supersonication for 10 min and centrifugation at 35,000 g for 20 min. The proteins in the membrane fractions were dissolved in lysis buffer B containing 7 M urea, 2.5 M thiourea, 2% CHAPS, 2.5% ASB-14, 50 mM DTT, 0.5% ampholyte, 1 mM PMSF, 2 mM EDTA and 20 mM Tris-HCl at pH 9.0. Protein concentrations were estimated by the Bradford assay [10].

### 4 The 2DE analysis

The *T. maritima* proteins (80 µg/gel) were rehydrated overnight with the commercial IPG strips with a linear range of pH 4.0–7.0 (18 cm). Electrofocusing was performed with IPGphor (Amersham Biosciences, Uppsala, Sweden) at 20°C with a linear voltage program from 50 V to 500 V for 1 h, 500 V to 1,000 V for 1 h, 1,000 V to 4,000 V for 1 h and 4,000 V to 8,000 V for 1 h, followed by 8,000 V for a total of 80,000 Vh. Prior to the second dimension electrophoresis, the electrofocused strips were reduced with DTT, alkylated with iodoacetamide and loaded onto 12% polyacrylamide gels (26×20 cm) using an Ettan DALT II system (Amersham Biosciences, Uppsala, Sweden). The separated proteins were visualized by a modified method of silver nitrate staining.

The 2DE gels were scanned with a Powerlook 2100XL scanner (UMAX, Taiwan, China). The image analysis was carried out by a combination of manual inspection and software analysis with the ImageMaster platinum software version 5.0 (Amersham Biosciences, Uppsala, Sweden). To count 2DE spots, triplicate gels were analyzed by ImageMaster; a miss-match rate of than less 5% between the parallel gels was allowed. The total 2DE spots from each sample were analyzed statistically. To compare the data for quantitative analysis, several key parameters in the image analysis were fixed as constants, such as smooth at 2, minimal area at 50 and saliency at 560. The relative spot volumes were normalized to the total spot volumes with a multiplication factor of 100.

### 5 Mass spectrometric identification of proteins

The 2DE spots were carefully excised and successively destained and dehydrated with acetonitrile. The treated gel slices were reduced with 10 mM DTT in 25 mM ammonium bicarbonate at 56°C for 1 h and alkylated with 55 mM iodoacetamide in 25 mM ammonium bicarbonate in the dark at room temperature for 45 min. Finally, the gel pieces were thoroughly washed with 25 mM ammonium bicarbonate in water/acetonitrile (50/50) and completely dried in a Speedvac. The proteins were digested in 10 µl of modified trypsin solution (1 ng/µl in 25 mM ammonium bicarbonate) at 37°C overnight. The digestion reaction was stopped by addition of 1 µl of 10% TFA.

The digested products were loaded on an Anchorchip target (Bruker Dalton, Bremen, Germany) and mixed with a matrix solution consisting of 4 mg/ml CHCA in 70% acetonitrile with 0.1% TFA. After drying at room temperature, the target was delivered to an UltraFlex MALDI TOF/TOF MS (Bruker Dalton, Bremen, Germany). The mass spectrometer was operated under a 25 kV accelerating voltage in the reflection mode with an m/z range of 700–4,000. Typically, 100 shots were collected per spectrum in MS mode and 400 shots in MS/MS mode with a mass tolerance of 100 ppm and MS/MS tolerance of 0.6 Da. The spectrum mass signals were processed using the FlexAnalysis 2.2 and BioTools 2.2 software and searched with MASCOT (http://www.matrixscience.com) against the NCBI nr database with bacteria as the taxonomy and with modifications, such as carboxymethylation, methionine oxidation and pyro-glutamine (N-term Q).

### 6 Western blot analysis

In all of the Western blot experiments, the primary antibodies were generated in our laboratory. Briefly, recombinant proteins cloned from the *T. maritima* genome, such as D-glyceraldehyde-3-phosphate dehydrogenase (gi|939978, GAPDH), 3-phosphoglycerate kinase/triosephosphate isomerase (gi|6226667, PGK/TIM), 3-phosphoglycerate kinase (gi|450686, PGK), pyruvate synthase porA (gi|3914401, PFOR), phosphomannomutase (gi|4981297, PMM), heat shock protein 60 (gi|4981018, Hsp60), fructose-bisphosphate aldolase (gi|4980771, FBA), were expressed in *E. coli* and purified by affinity chromatography. New Zealand rabbits were immunized with recombinant proteins in complete Freund's adjuvant (1∶1), followed by three boosts with the same amount of proteins in incomplete Freund's adjuvant (1∶1). The rabbit sera were collected and purified through protein A affinity chromatography (Bio-Rad, California, USA). According to the guidelines of the National Institutes of Health Guide for the Care and Use of Laboratory Animals, the mice had free access to food and tap water and been maintained on a 12 h light/dark cycle. The protocol of animal treatment was approved by the Animal Care and Welfare Committee in the Beijing Institute of Genomics, Chinese Academy of Sciences.

For SDS-PAGE Western blot analysis, 10 µg of *T. maritima* total protein was run on 12% polyacrylamide gels and transferred onto a polyvinylidene difluoride (PVDF) membrane. For 2DE Western blot analysis, 10 µg of *T. maritima* total protein was first isoelectrically focused on 7-cm IPG strips with 45,000 Vh and then run on 12% SDS-PAGE gels followed by electro-blotting onto a PVDF membrane. After treatment with blocking buffer containing 5% milk powder, the transferred PVDF membranes were incubated with the primary antibodies. The secondary antibody against rabbit IgG conjugated with horseradish peroxidase (HRP) was then incubated with the treated membranes followed by chemiluminescent detection using QuantECL instrument (Amersham Biosciences, Uppsala, Sweden).

### 7 Real-time PCR Analysis

Total RNA was isolated from the bacteria cultured at four different temperatures. The first-strand cDNA was synthesized by reverse transcriptase (Invitrogen, California) using the total RNA as the template. The primers were designed according to the corresponding gene sequences from *T. maritima*. The real-time PCR was carried out on an ABI PRISM 7300 system (Foster City, CA) with programmed parameters. The melting curves for each PCR reaction were carefully evaluated to avoid nonspecific amplifications in the PCR products. The *T. maritima* 16s rRNA was used for normalization. And the quantitative data was acquired according to the 2^−ΔΔCt^ method.

### 8 Statistical analysis

The average values of the parallel experiments are given as the means ± SD. The comparison of differences among the groups was performed with Student's *t* test. All tests were three-tailed, and the significance level was set at *p*<0.05.

## Results

### 1 The growth curves of *T. maritima* cultured at different temperatures

**Figure 1:**
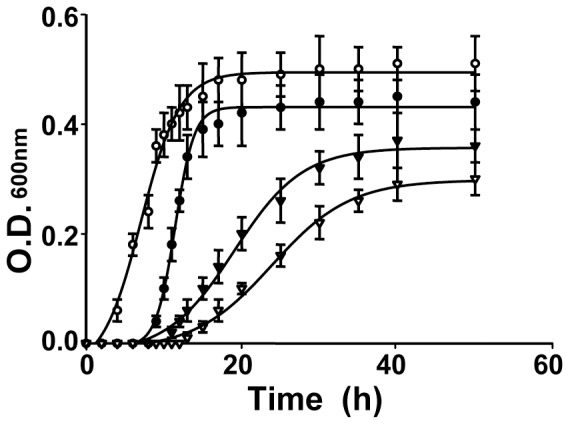
The growth curves of *T. maritima* at different culture temperatures. The sample collections and the curve fittings were described in “Materials and Methods”. The symbols of “▿”, “▾”, “○”and “•” represent the bacteria cultured at 60, 70, 80 and 90°C, respectively.

We re-tested several media types and conditions for culturing *T. maritima* based upon other reports. We finally developed a modified medium, called TMM, which is described in the Materials and Methods and was employed in this study [3], [11], [12]. The *T. maritima* bacteria were cultured at four different temperatures, 60, 70, 80 and 90°C, respectively. As shown Fig. 1, the growth rates within the temperature range were quite different, and the values of T_1/2_ for the log phase were estimated as 25 h at 60°C, 20 h at 70°C, 8 h at 80°C and 13 h at 90°C; while the T_end_ for the stationary phase were 50 h at 60°C, 45 h at 70°C,19 h at 80°C and 23 h at 90°C. The data suggested that *T. maritima* propagation was relatively poor at temperatures lower than 70°C and activated at temperatures around 80°C. The *T. maritima* cells at the T_1/2_ phase of different temperatures were then harvested for temperature-dependent proteome analysis.

### 2 Comparison of 2DE images for the *T. maritima* cytoplasmic proteins at different temperatures

**Figure 2:**
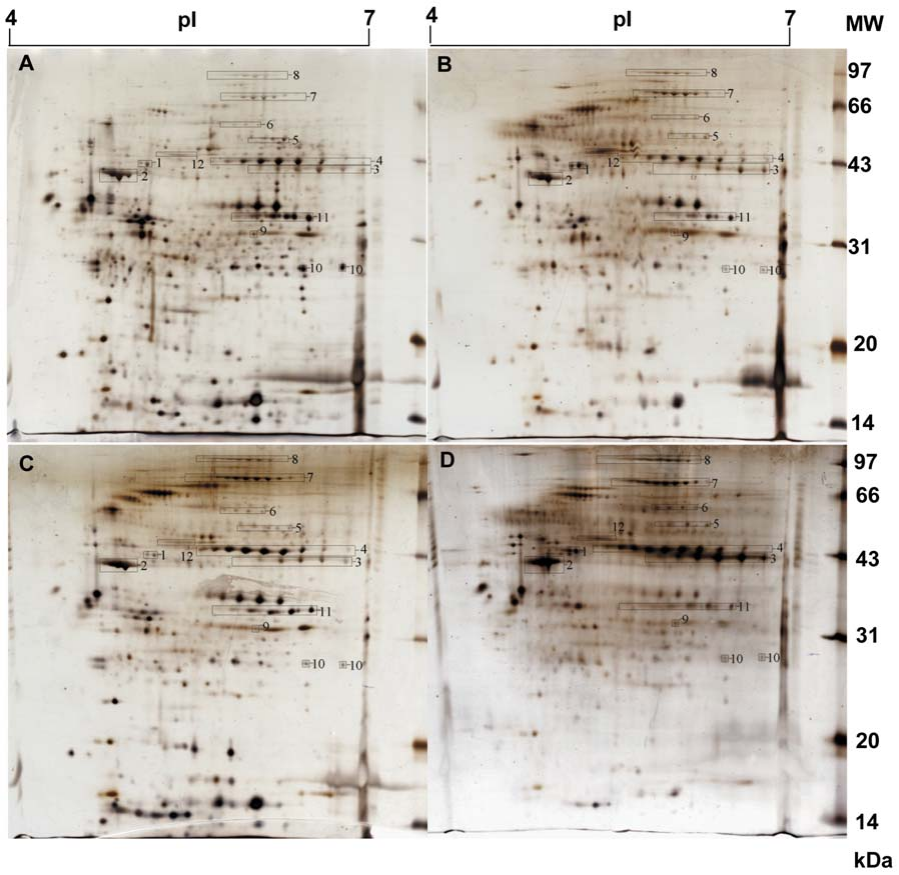
The 2-DE images of the cytoplasm proteins from *T. maritima* cultured at the four temperatures. A), B), C), and D) exhibit the 2-DE images for the bacteria cultured at 60, 70, 80 and 90°C, respectively.

We first examined the 2DE behavior of the *T. maritima* cytoplasmic proteins, and found that the bacterial proteins were mainly focused in the acidic region of the IPG strip. The narrow pH 4–7 strips was thus employed to achieve better resolution for these proteins. As shown in Fig. 2, the 2DE protein profiles showed satisfactory resolution and temperature-dependent modes. We observed that 1) the patterns of the 2DE spot distributions at the four temperatures were similar, and 2) the total number of 2DE spots per gel was dramatically decreased during temperature elevation, with 578±43 spots at 60°C, 441±15 at 70°C, 428±14 at 80°C and 377±16 at 90°C. To define the temperature-dependent 2DE spots, we adopted the high-quality image analysis with stringent criteria, 1) all the 2DE spot intensities were represented as relative 2DE spot volumes by normalizing the individual spot volumes to the sum of the total 2DE spot volumes in each gel; 2) setting the 2DE spot volumes from 60°C as the reference, differential spots were determined by the ratios of the individual spot volumes at certain temperature against the corresponding spot volume in the reference; 3) a change in spot volume of over three fold between any two samples was considered temperature-dependent and 4) the mode of temperature-dependent change in the 2DE spot was elicited through all four temperatures. Thus, 134 2DE spots were defined as temperature-dependent, with 58 up-, 37 down-regulated and 39 with a bell-shaped-regulation. Overall, 2DE spot intensities at 60 and 90°C were relatively lower than those at 70 and 80°C.

### 3 Comparison of 2DE images of the *T. maritima* membrane proteins at different temperatures

**Figure 3:**
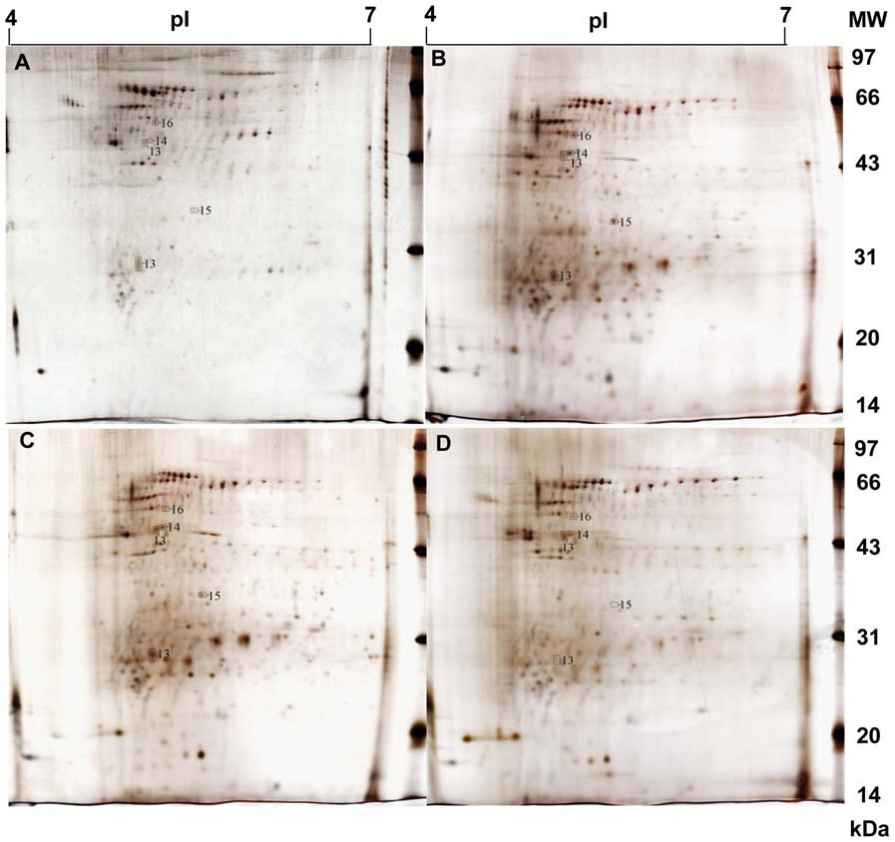
The 2-DE images of the membrane proteins from *T. maritima* cultured at the four temperatures. A), B), C), and D) exhibit the 2-DE images for the bacteria cultured at 60, 70, 80 and 90°C, respectively.

As illustrated in Fig. 3, the 2DE images for separation of the bacterial membrane proteins resulted in approximate 400 spots per gel, specifically, 413±16 at 60°C, 423±21 at 70°C, 432±23 at 80°C and 419±27 at 90°C. In contrast to the cytoplasmic proteins, the total numbers of 2DE spots from the membrane proteins seem insensitive to temperature changes. We adopted the same strategy above to screen the differential spots in the 2DE images and found a total of 90 2DE spots had altered volumes in response to temperature changes, including 12 up-, 51 down- and 27 bell-shaped-regulation.

### 4 Identification of the temperature-dependent proteins of *T. maritima*

For the identification of proteins using MALDI TOF/TOF MS, the stringent criteria were established to reduce false positives, 1) the identified protein with at least five matched peptides sequences; 2) the total sequence coverage over 15% and 3) at least one MS/MS spectrum. In the cytoplasm fraction, a total of 134 differential 2DE spots were analyzed by MALDI TOF/TOF MS, resulting in 109 identified as the bacterial proteins, including 41 unique proteins. In the membrane fraction, a total of 90 differential 2DE spots were analyzed, resulting in 55 identified as the bacterial proteins, including 37 unique proteins. Detailed information of the identified proteins is summarized in Table S1 and S2. The identification rates of the temperature-dependent spots were approximately 82% for the cytoplasmic proteins and 61% for the membrane proteins. There are three identified unique proteins, PFOR, histidine kinase (gi|4981919, HK) and a hypothetical protein (gi|4982076), were co-identified in both of the fractions.

### 5 Functional categorization of the temperature-dependent proteins

Using GO assignments from the InterProScan search (http://www.ebi.ac.uk/InterproScan/), we classified the 41 unique differential proteins in the cytoplasm into 11 categories as described in Fig. S1A. The proteins involved in central carbohydrate metabolism rank at the top of all categories (27%, 11/41). If redundant proteins were considered, 48.6% (53/109) of the identified proteins belong to this category. Of the 11 unique proteins related to carbohydrate metabolism, 8 are up-regulated with increasing temperatures, 2 are down-regulated and 1 has a bell-shaped-regulation profile. The whole 37 identified proteins in the membrane fraction were categorized into 11 groups as listed in Fig. S1B. In contrast to the cytoplasm proteins, the membrane proteins related to central carbohydrate metabolism, energy production and transcription are at similar percentages of 11% (4/37), 14% (5/37) and 16% (6/37), respectively.

**Figure 4:**
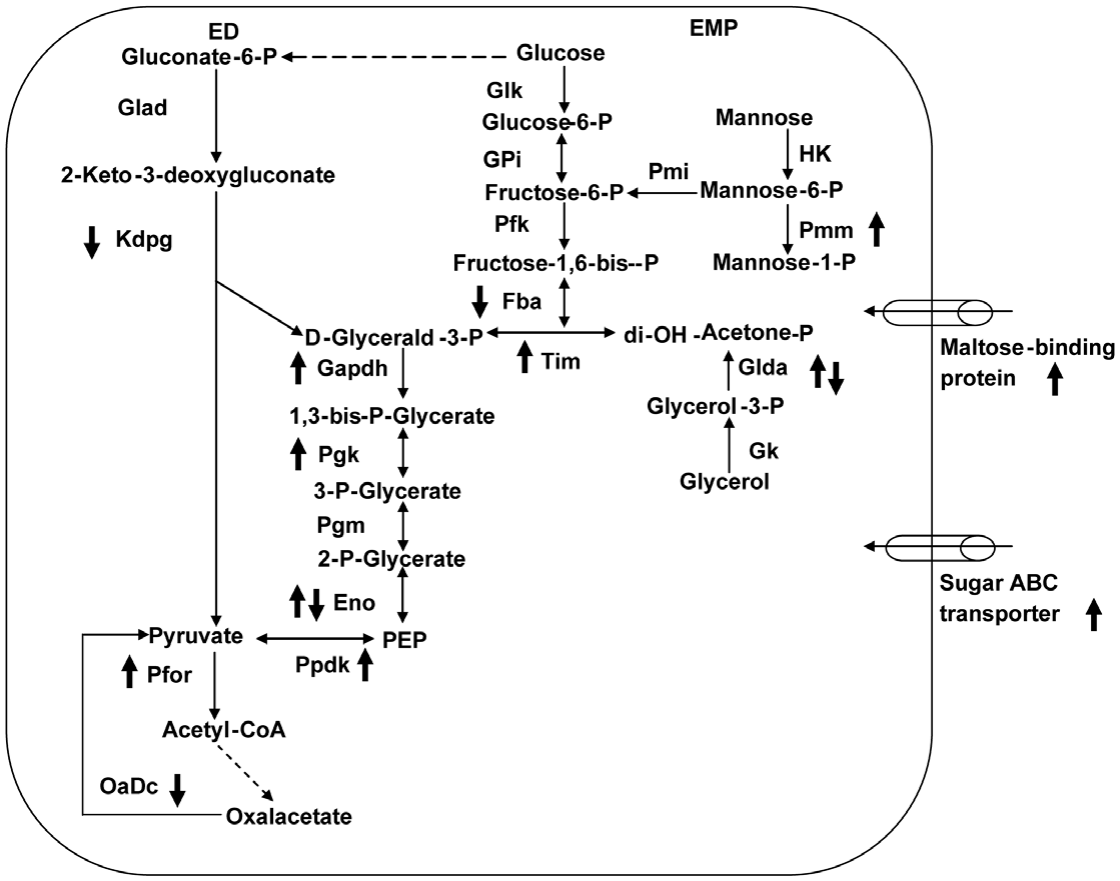
The possible network of central carbon metabolism in *T. maritima*. The symbols, “↑, ↓, and ↓↑,” indicate the protein abundances up-, down-, or bell-shaped-regulation response to temperature change. **Abbreviations: ED**, Entner–Doudoroff pathway; **EMP**, Embden–Meyerhof–Parnas pathway; **XynC**, family 10 xylanase XynC; **Glad**, gluconate dehydratase; **Kdpg**, 2-dehydro-3-deoxyphosphogluconate aldolase; **Glk**, glucokinase; **Gpi**, glucose-6-phosphate isomerase; **Pfk**, phosphofructokinase; **Fba**, fructose-1,6-bisphosphate aldolase; **HK**, hexokinase; **Pmi**, phosphomannoisomerase; **Pmm**, phosphomannomutase; **Tim**, triosephosphate isomerase; **Glda**, glycerol dehydrogenase; **Gk**, glycerol kinase; **Gapdh**, D-glyceraldehyde-3-phosphate dehydrogenase; **Pgk**, 3-phosphoglycerate kinase; **Pgm**, phosphoglycerate mutase; **Eno**, enolase; **Ppdk**, pyruvate,orthophosphate dikinase; **Pfor**, pyruvate synthase subunit porA; **OaDc**, oxaloacetate decarboxylase; **CoA**, coenzyme A; **PEP**, phosphoenolpyruvate.

**Figure 5:**
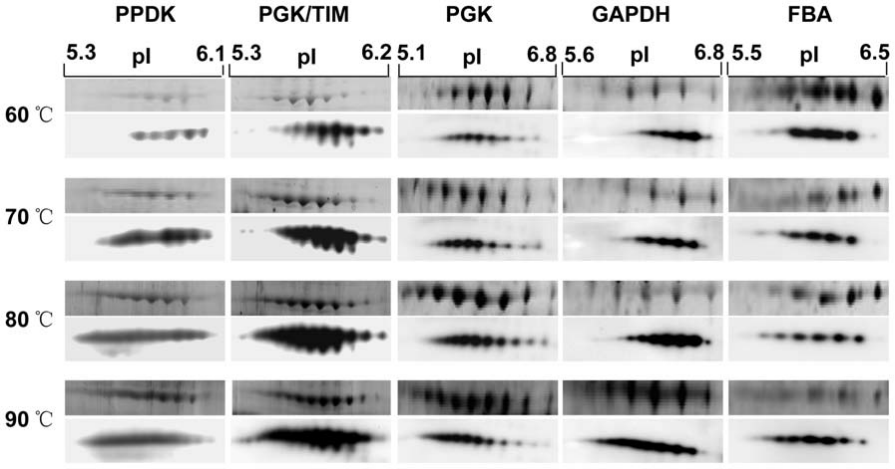
Close-up images of 2-DE and 2-DE Western blots of the five typical proteins with iso-spots and the significant changes in spot volumes responding to temperature. For each protein, the upper panels are the 2-DE images and the lower panels are the 2-DE Western blot images.

If all of the identified proteins, excluding unique ones, are functionally categorized, 58 of the proteins belonged to central carbohydrate metabolism, approximately 35% (58/164). The highest occupancy of carbohydrate metabolism proteins implies that these proteins in *T. maritima* are quite sensitive to environmental thermal stress. All of the temperature-dependent proteins related to central carbohydrate metabolism are summarized in Table 1. Furthermore, we specifically identified the locations of these proteins in the metabolic pathways of central carbohydrate metabolism. As demonstrated in Fig. 4, of 15 such unique proteins, 12 are widely distributed along the pathways, including 8 in the traditional EMP and ED pathways. We also examined the temperature-dependent modes of these unique proteins. The majority of these proteins (67%, 10/15) exhibit up-regulation in response to increasing temperature, while 20% (3/15) are down-regulated and 13% (2/15) have a bell-shaped-regulation profile. It is worth noting that some up-regulated proteins in this category exist in multiple 2DE spots. For instance, five proteins in the cytoplasmic fraction, pyruvate orthophosphate dikinase (PPDK), FBA, GADPH, PGK/TIM and PGK, were identified in several iso-spots in the pH 5–7 range and display an increased spot volume during temperature elevation (Fig. 5).

**Table 1:** The temperature-dependent proteins of *T.maritima* related to central carbohydrate metabolism and identified by MALDI TOF/TOF MS.

Spot #	Gi number	Protein name	MW he./Exp.	pI The./Exp.	Peptide matched	Sequence coverage	MASCOT score	Peptides identified by MS/MS
**Up-regulated cytoplasm proteins**								
**1**	gi|3914401	Pyruvate synthase subunit porA	44.1/40.3	5.01/5.50	29	62%	261	IWMFRPFPK EQLQELLNGR YVVDELREEGYK
**2**	gi|1850900	Periplasmic maltose-binding protein	43.0/40.0	4.93/5.05	17	34%	188	IYLADPR VIAMEFLTNFIAR QIDEEYGGEVR ETPQGLDVTDIGLANEGAVK YGIPVEVQYVDFGSIK
**3**	gi|939978	D-glyceraldehyde-3-phosphate dehydrogenase	36.4/42.3	6.23/6.76	17	61%	250	VLDLPHKDLR DLGVDFVIESTGVFR VASWYDNEYGYSNR FGIVSGMLTTVHSYTNDQR
**4**	gi|450686	3-Phosphoglycerate kinase	42.2/43.0	5.63/6.80	18	51%	179	AVEELKEGEVLLLENTR VDFNVPVKDGVVQDDTR VILLSHLGRPK GEPSPEFSLAPVAKR
**5**	gi|4981297	Phosphomannomutase	44.3/56.4	6.00/6.03	11	26%	98	IFVRPSGTEPK LINFKFEDVDR LKVYIHVR
**6**	gi|6226667	Bifunctional phosphoglycerate kinase, Triosephosphate isomerase	71.7/70.4	5.68/6.15	32	49%	267	VILLSHLGRPK VVIAYEPVWAIGTGR VATPQQAQEVHAFIR AVEELKEGEVLLLENTR KFGAEGIGLCR FGDPNNPLLVSVR VDFNVPVKDGVVQDDTR
**7**	gi|4980770	Pyruvate, orthophosphate dikinase	98.4/95.6	5.74/6.10	42	44%	308	FGDPNNPLLVSVR
**8**	gi|6730110	Triosephosphate Isomerase	28.7/32.3	5.60/6.01	8	39%	95	IFKEDDEFINR VATPQQAQEVHAFIR DIDGGLVGGASLKESFIELAR VVIAYEPVWAIGTGR
**Down-regulated cytoplasm proteins**								
**9**	gi|15642841	2-Dehydro-3-deoxyphosphogluconate aldolase	22.4/28.2	6.92/4.94	9	46%	128	GAIIGAGTVTSVEQCR
**10**	gi|4980771	Fructose-bisphosphate aldolase	34.9/34.5	5.85/6.50	15	49%	214	FKGEAQLDFER LSVPVALHLDHGR LSVPVALHLDHGRDFK
**Bell-shaped-regulated cytoplasm proteins**								
**11**	gi|4980929	Glycerol dehydrogenase	40.0/44.1	5.17/5.35	9	37%	110	YVQGAGAINILEEELSR NPDVVLVDTEIVAKAPAR VAIGVLASLFLTDKPR
**Up-regulated membrane proteins**								
**12**	gi|15642889	Sugar ABC transporter	36.1/50.2	5.15/5.12	5	17%	59	
**13**	gi|3914401	Pyruvate synthase subunit porA	44.1/50.5	5.01/5.17	11	33%	150	LLTLEEVTKDKPIR
**Down-regulated membrane protein**								
**14**	gi|4980620	Oxaloacetate decarboxylase	53.3/27.5	5.63/4.98	10	22%	59	
**Bell-shaped-regulated membrane proteins**								
**15**	gi|6226608	Enolase	46.9/53.4	4.93/5.25	9	24%	92	

Notes: The.:Theoretical value; Exp.:Experiment value.

Considering the correlation in the abundances of carbohydrate metabolism enzymes and energy generation, it is not surprising that in this case, the proteins involved in energy generation may follow similar trends as carbohydrate metabolism. As a matter of fact, three enzymes participating in ATP metabolism were detected in the temperature-dependent spots of membrane fraction, ATP synthase F0 (gi|4982186), F1F0-ATPase subunit (gi|2687836) and ATP synthase F1 subunit (gi|4982183) (Table S2).

### 6 Validation of temperature-dependent proteins involved in central carbohydrate metabolism pathways in *T. maritima*

It is necessary to do validate proteomic observations since most of the differential proteins in central carbohydrate metabolism were identified as multiple iso-spots. We took two validation approaches. Firstly, the temperature-dependent proteins were testified by Western blot. Secondly, the correspondent mRNAs were quantitatively estimated through real-time PCR.

**Figure 6:**
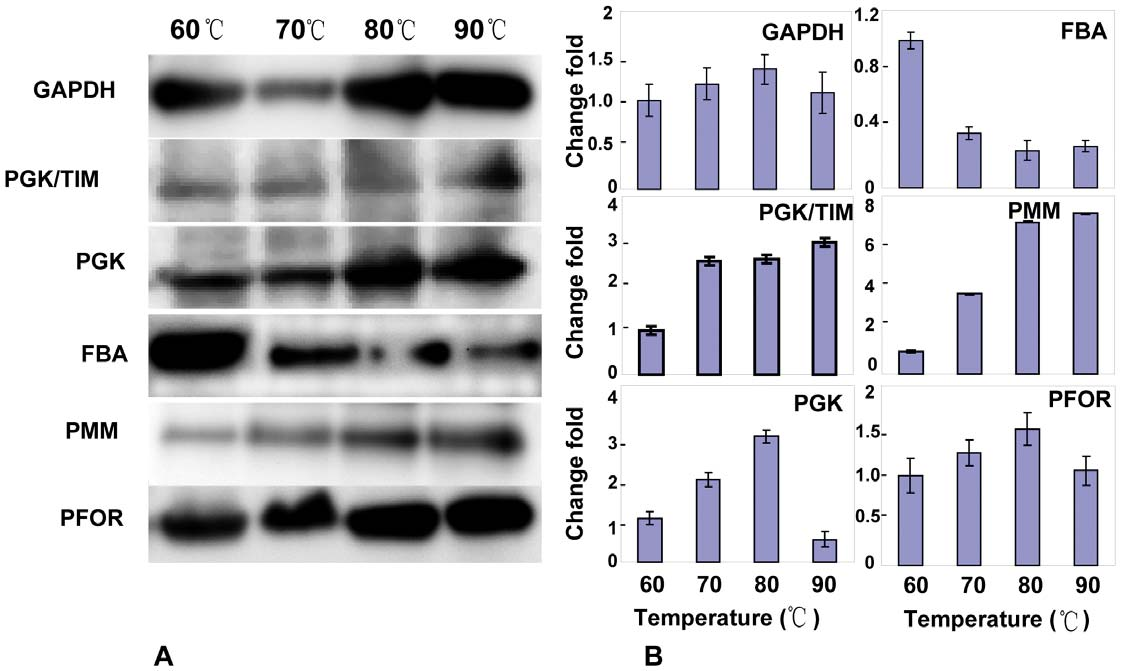
The temperature-dependent expression of the *T. maritima* carbohydrate metabolism related genes validated by Western blot and real-time PCR. A) The temperature-dependent proteins of central carbohydrate pathways in *T. maritima* were validated by Western blot. The six temperature-dependent proteins in *T. maritima* were specifically recognized by their correspondent antibodies at the four temperatures. B) The temperature-dependent expression of the T. maritima carbohydrate metabolism related genes were validated by real-time PCR. The experimental procedure and the statistic estimation for the parallel runs were described in “Material and Methods”.

A total of six temperature-dependent proteins were selected, 5 up-regulated and 1 down-regulated. As depicted in Fig. 5, the intensity changes of the immuno-reactive bands basically agreed with the 2DE proteomic observations. To further evaluate the pattern change of 2DE iso-spots during the temperature shift, a 2DE Western blot was employed, and the ECL images shown in Fig. 6A produced similar information as the proteomic analysis, in which the iso-spots displayed increased spot volumes during temperature increase.

An intriguing question is whether changes in protein abundance are correlated with their mRNA levels for the same functional category? The values of the cycle thresholds for all the six genes exhibited temperature-responsive modes, which were similar to those observed in the proteomic analysis (Fig. 6B); however, the mRNAs of the three up-regulated genes, *GAPDH*, *PGK* and *PFOR*, were found at decreased levels at 90°C.

### 7 Comparing the expression patterns of temperature-dependent proteins in *T. maritima* and *T. Tengcongensis*

**Figure 7:**
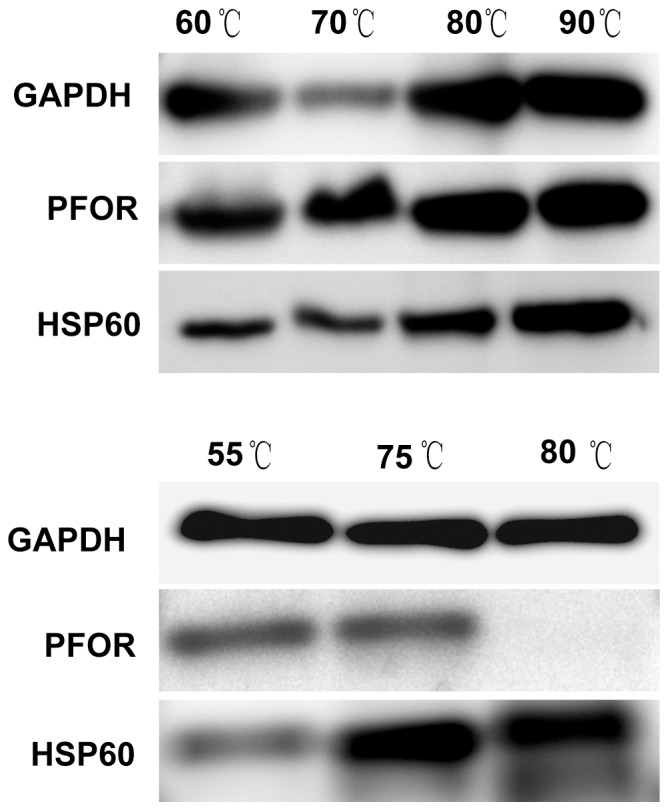
The expression pattern of temperature-dependent proteins in *T. maritima* and *T. tengcongensis*. The upper panel is the proteins from *T.maritima* and the lower panel is the proteins from *T. tengcongensis*.

In our previous investigation, the temperature-dependent proteomes were characterized in *T. tengcongensis* during exposure to three different temperatures [13]. This prompted the question of whether the temperature-dependent proteins are commonly shared by *T. tengcongensis* and *T. maritima*? We detected the abundances of GAPDH and PFOR in *T. tengcongensis* at three temperatures using Western blot with the antibodies specifically against this bacterial proteins (Fig. 7). Compared to *T. maritima*, the GAPDH abundance was not significantly changed within the temperature range of 55 to 80°C, whereas the PFOR abundance was sharply reduced at 80°C. Obviously, the abundance response of the two proteins to temperature in *T. maritima* were different from that found in *T. tengcongensis*, suggesting that the biochemical roles of the two proteins are dependent on the complicity of metabolic networks in each thermophile, at least at high temperature.

## Discussion

The changes in protein abundances in *T. maritima* during thermal stress were characterized for the first time in this study. The evidence presented here revealed that a large number of enzymes that participate in central carbohydrate metabolism were effectively regulated by temperature in this bacterium. This conclusion disagrees with a previous report on the transcriptional responses to thermal stress in the same bacteria. In that study, *T. maritima* was cultured and harvested at seven time points over period of 90 min following temperature shift from 80 to 90°C, and its transcriptional responses were analyzed using a targeted cDNA microarray, which consisted of 20% of the bacterial genome [9]. Marybeth *et al.* observed that most heat-shock genes were expressed at low levels at 80°C but significantly induced after the 10°C temperature increase, such as the *DnaK* chaperone machinery (gi|15643613, gi|15643612 and gi|15643141), *GroESL* (gi|15643271 and gi|15643272) and *HrcA* (gi|15643614). Meanwhile, several members of the SOS regulon, *lexA/dinR* (gi|15643840), *recA* (gi|15644602), *ruvA* and *ruvB* (gi|15642939 and gi|15644476) and *uvrB* (gi|15644506) were also positively induced by the elevated temperature, leading to the hypothesis that the *T. maritima* SOS regulon played a key role in the regulation of DNA repair at high temperatures [9]. The temperature-dependent proteins of *T. maritima* identified in this study contain only one traditional chaperone protein, HSP60, and many metabolism-related proteins, but no SOS regulon proteins. Why do the transcriptomic and the proteomic data give such different answers to the same question? We attribute the conflicting results to several factors. First, the sensitivity of the methodology is a fundamental factor. Compared with the fluorescent signals monitored on cDNA chips, 2DE has a relatively low sensitivity for protein detection. On the other hand, the mRNA abundance does not completely reflect the information held in protein abundances, because translation does not always coincide with transcription, and the half-lives of mRNAs are different to the half-lives of protein. We also realize that the size of the DNA microchip, that is how many corresponding genes are present on chip, may affect the screening results and lead to a false deduction. More importantly, the data reported by the Marybeth group was obtained from the transcriptional responses to a short 90-min period when the culture temperature increased by 10°C, whereas our study focused on the translational response at log phase growth at four temperatures, in which the bacteria were in a stable adaptive status. All of the data acquired from different analyses, either transcriptional or translational responses, therefore provide the gene expression information of *T. maritima* in responding to temperature changes.

The density of genes involved in central carbohydrate metabolism in the *T. maritima* genome is significantly higher than in many other bacteria, approximately 35 per Mb of sequence, whereas there are about 15 per Mb of sequence in the genomes of *Escherichia coli* and *T. tengcongensis* [14]. In the EMP pathway, allosteric regulation at the protein level plays an essential role in bacteria, while most ED genes are regulated by environmental changes in archaeon [15]. Recently, Zaparty *et al.* employed DNA microarrays to unravel the role of transcriptional regulation in central carbohydrate metabolism in *Thermoproteus tenax* [16]. The main control point for central carbohydrate metabolic pathways in archaea was proposed at the site of glyceraldehyde-3-phosphate (GAP), through which the changes in expression of GAP-related genes, either at the mRNA or protein level, were mediated by environmental stimuli. GAPDH, non-phosphorylating glyceraldehyde-3-phosphate dehydrogenase (GAPN) and glyceraldehyde-3-phosphate dehydrogenate oxidoreductase (GAPOR) were likely the key members. Our data for the temperature-dependent proteome provides another clue for the regulation of central carbohydrate metabolism at the protein level in eubacteria responding to thermal stress. As proposed in Fig. 4, a number of proteins up-regulated due to elevated temperatures in *T. maritima* were located downstream of glyceraldehyde-3-phosphate, such as GAPDH, 3-phosphoglycerate kinase and enolase. Therefore, the fork in EMP and ED pathways may also be a regulatory site in eubacteria, at least in *T. maritima*.

Our early results revealed that the electrophoretic images of the *T. tengcongensis* proteins cultured at 55 and 75°C showed similar patterns, whereas the number of electrophoretic spots from the extracts of cells at 80°C were dramatically reduced [13]. This phenomenon was corroborated in *T. maritima* as well. As shown in Fig. 2, the number of cytoplasmic 2DE spots from the *T. maritima* extracts at 90°C was significantly lower than those from extracts of the other three culture temperatures. Furthermore, we analyzed and identified several different spots that appeared in the 2DE images of the two thermophiles, and compared the abundance changes of temperature-dependent proteins between them. For instance, seven proteins were found as up-regulated during temperature elevation in *T. tengcongensis*, such as dipeptidyl aminopeptidase (TTE1551), histidinol phosphatase (TTE1963), signal recognition GTPase (TTE1462), HSP10 (TTE0579), HSP60 (TTE0580), ABC-type transporter SufB (TTE2672) and a hypothetical protein (TTE0418) [13]. However, only one protein, HSP60, was shared with the up-regulated proteins of *T. maritima*. We also compared the three typically temperature-dependent proteins from the two thermophiles. As illustrated in Fig. 7, except for HSP60, the other two proteins, GAPDH and PFOR, exhibited differential responses to temperature changes. GAPDH and PFOR are the rate-limited enzymes in glycolysis in thermophiles. Theoretically speaking, the abundance changes of the two enzymes are expected to trend similarly once glycolysis is activated. In *T. maritima* both enzymes, GAPDH and PFOR, underwent almost identical changes at mRNA and protein levels when responding to temperature, whereas, in *T. tengcongensis* GAPDH remained consistent in its abundance among the temperature ranges examined and PFOR dropped in abundance at 80°C. Based on the genomes of the two thermphiles, *T. maritima* has a complete tricarboxylic acid cycle (TCA), while *T. tengcongensis* has a reduced TCA. It is suspected that GAPDH and PFOR may perform different roles in carbohydrate metabolism in each thermophile. Our proteomic data appears to support this hypothesis.

In summary, we adopted an electrophoresis-based proteomic approach in combination with molecular biology to survey the proteomic responses of *T. maritima* at different temperatures. On the basis of the 2DE and MS data, higher percentiles of proteins participating in central carbohydrate metabolism pathways were identified in the up-regulated proteins responding to increased temperature. The experiments of real-time PCR and immunoblots to measure the correspondent gene or protein abundances further provided supportive evidences to the proteomic observations. We therefore come to the conclusion that central carbohydrate metabolism pathways of *T. maritima* are likely activated at higher temperature because several key enzymes in the pathways exhibit increased abundances due to rising temperature. Since the mRNA abundance changes for the genes encoding the temperature-dependent proteins were almost synchronized with the protein abundance responses to temperature, we also postulate that *T. maritima* may contain some temperature-sensitive regulators that control gene transcription and translation once the environmental temperature changes.

## Supporting Information

Figure S1 The classification pattern of unique differential proteins in *T. maritima*. Fig. S1A) exhibit the 41 unique differential proteins in the cytoplasm fraction were categorized into 11 groups, Fig. S1B) exhibit the 37 unique differential proteins in the membrane fraction were categorized into 11 groups.
(TIF)

Table S1 The results of identification and classification of the soluble temperature-dependent proteins.
(DOC)

Table S2 The results of identification and classification of the membrane temperature-dependent proteins.
(DOC)
