# Supplementary figures and images for: The Temperature Dependent Proteomic Analysis of Thermotoga maritima
Source: PLoS One. 2012 Oct 5;7(10):e46463. doi: 10.1371/journal.pone.0046463 (PMC3465335; doi:10.1371/journal.pone.0046463)

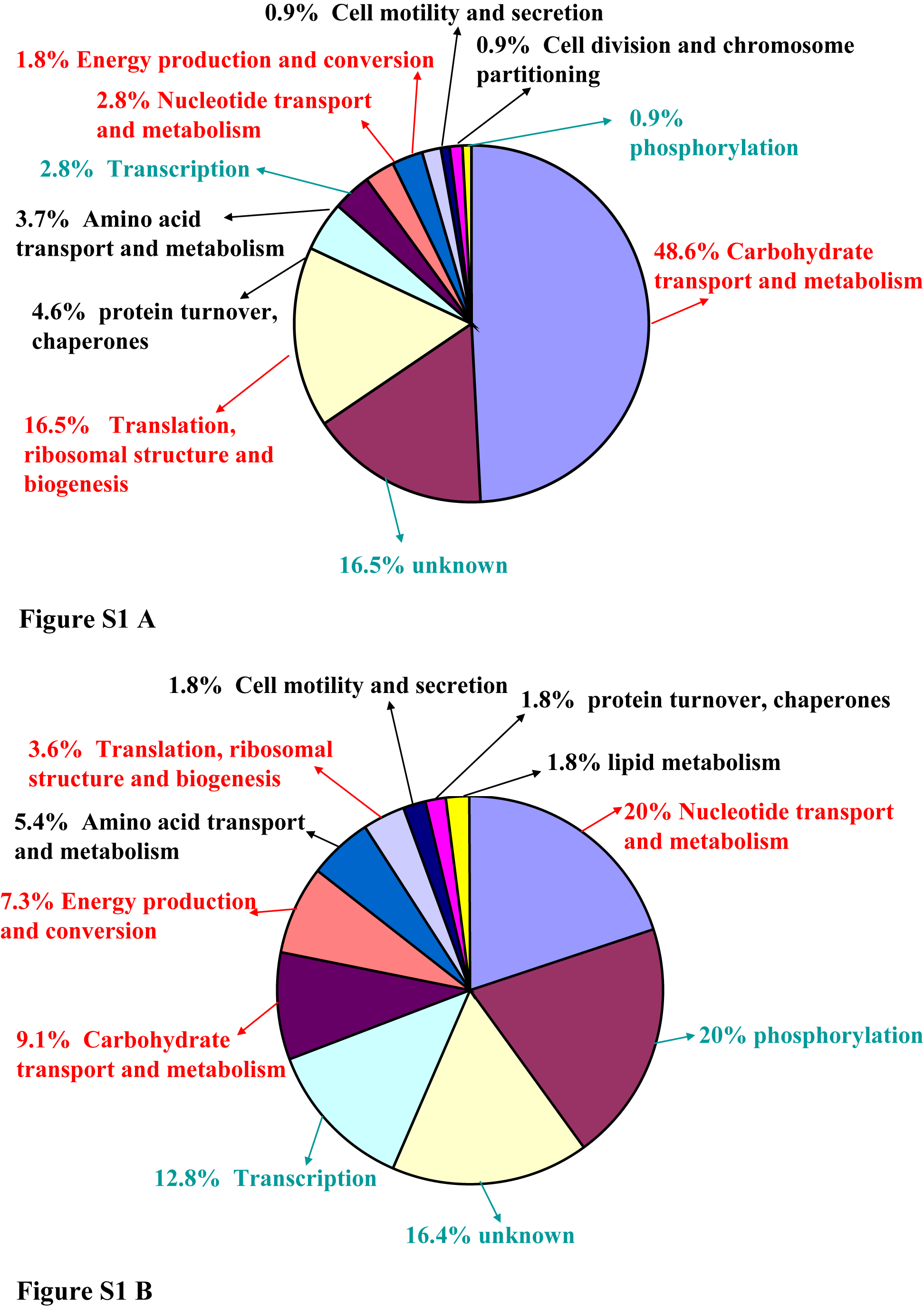

Supplement: Figure S1 — The classification pattern of unique differential proteins in T. maritima. Fig. S1A) exhibit the 41 unique differential proteins in the cytoplasm fraction were categorized into 11 groups, Fig. S1B) exhibit the 37 unique differential proteins in the membrane fraction were categorized into 11 groups. (TIF) [file pone.0046463.s001.tif]
