# Supplementary material for: The Temperature Dependent Proteomic Analysis of Thermotoga maritima
Source: PLoS One. 2012 Oct 5;7(10):e46463. doi: 10.1371/journal.pone.0046463 (PMC3465335; doi:10.1371/journal.pone.0046463)
Supplement: Table S1 — The results of identification and classification of the soluble temperature-dependent proteins. (DOC) [file pone.0046463.s002.doc]

**Table S1. The results of i**dentification and classification of the soluble temperature-dependent proteins

| **Spot #** | **Gi number** | **Protein name** | **MW**  **The./Exp.** | **pI**  **The./Exp.** | **Peptide matched** | **Sequence coverage** | **MASCOT score** | **Peptides identified by MS/MS** | **Function classification** |
| --- | --- | --- | --- | --- | --- | --- | --- | --- | --- |
| **Up-regulated** | | |  |  |  |  |  |  |  |
| **1** | gi|3914401 | Pyruvate synthase subunit porA | 44.1/40.3 | 5.01/5.50 | 29 | 62% | 261 | IWMFRPFPK  EQLQELLNGR  YVVDELREEGYK | Carbohydrate transport and metabolism |
| **2** | gi|1850900 | Periplasmic maltose-binding protein | 43.0/40.0 | 4.93/5.05 | 17 | 34% | 188 | IYLADPR  VIAMEFLTNFIAR  QIDEEYGGEVR  ETPQGLDVTDIGLANEGAVK  YGIPVEVQYVDFGSIK | Carbohydrate transport and metabolism |
| **3** | gi|939978 | D-glyceraldehyde-3-phosphate dehydrogenase | 36.4/42.3 | 6.23/6.76 | 17 | 61% | 250 | VLDLPHKDLR  DLGVDFVIESTGVFR  VASWYDNEYGYSNR  FGIVSGMLTTVHSYTNDQR | Carbohydrate transport and metabolism |
| **4** | gi|450686 | 3-Phosphoglycerate kinase | 42.2/43.0 | 5.63/6.80 | 18 | 51% | 179 | AVEELKEGEVLLLENTR  VDFNVPVKDGVVQDDTR  VILLSHLGRPK  GEPSPEFSLAPVAKR | Carbohydrate transport and metabolism |
| **5** | gi|4981297 | Phosphomannomutase | 44.3/56.4 | 6.00/6.03 | 11 | 26% | 98 | IFVRPSGTEPK  LINFKFEDVDR  LKVYIHVR | Carbohydrate transport and metabolism |
| **6** | gi|6226667 | Bifunctional phosphoglycerate kinase, Triosephosphate isomerase | 71.7/70.4 | 5.68/6.15 | 32 | 49% | 267 | VILLSHLGRPK  VVIAYEPVWAIGTGR  VATPQQAQEVHAFIR  AVEELKEGEVLLLENTR  KFGAEGIGLCR  FGDPNNPLLVSVR  VDFNVPVKDGVVQDDTR | Carbohydrate transport and metabolism |
| **7** | gi|4980770 | Pyruvate,orthophosphate dikinase | 98.4/95.6 | 5.74/6.10 | 42 | 44% | 308 | FGDPNNPLLVSVR | Carbohydrate transport and metabolism |
| **8** | gi|441103 | Triosephosphate Isomerase | 28.7/32.3 | 5.60/6.01 | 8 | 39% | 95 | IFKEDDEFINR  VATPQQAQEVHAFIR  DIDGGLVGGASLKESFIELAR  VVIAYEPVWAIGTGR | Carbohydrate transport and metabolism |
| **9** | gi|226378 | Elongation factor Tu | 44.6/66.2 | 5.31/5.63 | 24 | 68% | 251 | DLLSQYGYPGDEVPVIR  ELDEGIAGDNVGCLLR  GITINITHVEYETEKR | Translation, ribosomal structure and biogenesis |
| **10** | gi|4981728 | Flagellar protein FliS | 15.7/35.5 | 6.41/4.67 | 3 | 35% | 65 |  | Translation, ribosomal structure and biogenesis |
| **11** | gi|12230736 | Trigger factor | 49.9/59.5 | 4.79/4.83 | 19 | 44% | 154 |  | Translation, ribosomal structure and biogenesis |
| **12** | gi|4981018 | GroEL protein | 58.1/61.2 | 5.05/5.31 | 23 | 42% | 330 | AAVEEGIVPGGGVTLLR  AQIEETTSEYEKETLQER  EIELEDKFENLGAQLVK | protein turnover, chaperones |
| **13** | gi|4980883 | NADH oxidase | 47.7/54.1 | 5.78/6.29 | 16 | 41% | 137 |  | Energy production and conversion |
| **14** | gi|2104497 | Chain B, Ftsz, T7 Mutated, Domain Swapped | 38.6/39.0 | 5.68/5.81 | 13 | 39% | 130 |  | Cell division and chromosome partitioning |
| **15** | gi|4981682 | branched chain amino acid ABC transporter | 40.2/35.4 | 5.27/4.64 | 13 | 38% | 109 |  | Amino acid transport and metabolism |
| **16** | gi|4982076 | Hypothetical protein | 47.8/54.0 | 5.79/6.19 | 14 | 31% | 107 | EVLEPIFGISNPR  RVPEDVVESLDVNILQR | Unknown |
| **Down-regulated** | | |  |  |  |  |  |  |  |
| **17** | gi|15642841 | 2-Dehydro-3-deoxyphosphogluconate aldolase | 22.4/28.2 | 6.92/4.94 | 9 | 46% | 128 | GAIIGAGTVTSVEQCR | Carbohydrate transport and metabolism |
| **18** | gi|4980771 | Fructose-bisphosphate aldolase | 34.9/34.5 | 5.85/6.50 | 15 | 49% | 214 | FKGEAQLDFER  LSVPVALHLDHGR  LSVPVALHLDHGRDFK | Carbohydrate transport and metabolism |
| **19** | gi|7388035 | Orotate phosphoribosyltransferase | 20.6/23.0 | 6.75/6.52 | 5 | 34% | 71 |  | Nucleotide transport and metabolism |
| **20** | gi|4981151 | Nucleotide sugar epimerase | 39.2/36.2 | 5.76/4.93 | 7 | 17% | 67 |  | Nucleotide transport and metabolism |
| **21** | gi|18203584 | Uracil phosphoribosyltransferase | 23.4/27.1 | 5.62/5.81 | 6 | 40% | 112 | INDKDIVVVPILR  LNDHGYIIPGLGDAGDR  EITLLLAYEATR  ELLREITLLLAYEATR | Nucleotide transport and metabolism |
| **22** | gi|42559545 | Redox-sensing transcriptional repressor rex 2 | 23.3/26.1 | 5.76/6.12 | 17 | 59% | 228 | FLEINPDLVR | Transcription |
| **23** | gi|4980946 | Transcriptional regulator, GntR family | 25.2/18.3 | 5.36/4.67 | 6 | 31% | 55 |  | Transcription |
| **24** | gi|4981698 | Oligopeptide ABC transporter | 57.8/36.4 | 4.84/4.38 | 19 | 40% | 190 | LALETGEIDVAYR  TIVINFYENASTLR | protein turnover, chaperones |
| **25** | gi|4981403 | Glutaredoxin-related protein | 25.4/35.3 | 4.85/5.12 | 12 | 56% | 152 | FGVSSVPHIVVNR  ISVFVTPTCPYCPR  FGVSSVPHIVVNR  ISVFVTPTCPYCPR  EFINEVLR  LQSLEEPIR | Energy production and conversion |
| **26** | gi|4981224 | Purine-binding chemotaxis protein | 17.0/20.0 | 5.15/4.75 | 5 | 23% | 58 | HFVEGVINLR  TKDVEVGFLVDR | Cell motility and secretion |
| **27** | gi|4980974 | Conserved hypothetical protein | 17.0/31.0 | 5.04/5.12 | 5 | 23% | 58 |  | Unknown |
| **28** | gi|15642959 | Hypothetical protein | 13.4/12.0 | 4.85/4.90 | 6 | 35% | 60 |  | Unknown |
| **29** | gi|4981420 | conserved hypothetical protein | 20.2/21.2 | 5.87/6.41 | 6 | 37% | 66 |  | Unknown |
| **Bell-shaped-regulated** | | |  |  |  |  |  |  |  |
| **30** | gi|4980929 | Glycerol dehydrogenase | 40.0/44.1 | 5.17/5.35 | 9 | 37% | 110 | YVQGAGAINILEEELSR  NPDVVLVDTEIVAKAPAR  VAIGVLASLFLTDKPR | Carbohydrate transport and metabolism |
| **31** | gi|6226720 | Anthranilate synthase component 1 | 52.3/50.3 | 5.70/5.34 | 9 | 25% | 60 |  | Translation, ribosomal structure and biogenesis |
| **32** | gi|7387699 | Glutamyl-tRNA(Gln) amidotransferase subunit A | 52.7/37.4 | 7.60/5.61 | 8 | 15% | 58 |  | Translation, ribosomal structure and biogenesis |
| **33** | gi|4982212 | Electron transfer protein | 31.4/31.8 | 5.37/5.67 | 9 | 35% | 121 | EARPGQFVVIR  IPLTVADTKPEEGLFR | Translation, ribosomal structure and biogenesis |
| **34** | gi|4980873 | Conserved hypothetical protein | 16.2/18.3 | 5.41/5.62 | 9 | 65% | 116 |  | Translation, ribosomal structure and biogenesis |
| **35** | gi|4982087 | Dihydrodipicolinate synthase | 32.5/31.2 | 5.91/5.82 | 11 | 50% | 148 | TGVNVLPETAAR  TDLGIVVYNVPGR | Amino acid transport and metabolism |
| **36** | gi|4981919 | Sensor histidine kinase | 89.0/32.2 | 5.96/5.63 | 11 | 16% | 63 |  | phosphorylation |
| **37** | gi|15644109 | Hypothetical protein TM1357 | 52.6/49.4 | 5.02/4.75 | 9 | 16% | 66 |  | Unknown |
| **38** | gi|4981519 | Hypothetical protein | 13.5/14.9 | 5.60/5.42 | 3 | 41% | 109 | NTKPEMLGLLPLSICHK | Unknown |
| **39** | gi|4980728 | Conserved hypothetical protein | 17.9/17.4 | 5.39/6.05 | 11 | 76% | 268 | IKKPPISLEVDGLR  TLVVNPGEACGYLSGR | Unknown |
| **40** | gi|4980666 | Conserved hypothetical protein | 17.9/12.3 | 9.78/4.91 | 4 | 33% | 102 |  | Unknown |
| **41** | gi|15642868 | Hypothetical protein | 14.4/14.8 | 8.89/5.50 | 3 | 39% | 53 |  | Unknown |

Notes: The.:Theoretical value; Exp.:Experiment value.
