# Supplementary material for: The Temperature Dependent Proteomic Analysis of Thermotoga maritima
Source: PLoS One. 2012 Oct 5;7(10):e46463. doi: 10.1371/journal.pone.0046463 (PMC3465335; doi:10.1371/journal.pone.0046463)
Supplement: Table S2 — The results of identification and classification of the membrane temperature-dependent proteins. (DOC) [file pone.0046463.s003.doc]

**Table S2 The results of i**dentification and classification of the membrane temperature-dependent proteins

| **Spot #** | **Gi number** | **Protein name** | | **MW**  **The./Exp.** | **pI**  **The./Exp.** | | **Peptide matched** | **Sequence coverage** | | | **MASCOT score** | **Peptides identified by MS/MS** | **Function classification** |
| --- | --- | --- | --- | --- | --- | --- | --- | --- | --- | --- | --- | --- | --- |
| **Up-regulated** | | | |  | |  | | |  | | |  |  |
| **1** | gi|15642889 | Sugar ABC transporter | | 36.1/50.2 | 5.15/5.12 | | 5 | 17% | | | 59 |  | Carbohydrate transport and metabolism |
| **2** | gi|3914401 | Pyruvate synthase subunit porA | | 44.1/50.5 | 5.01/5.17 | | 11 | 33% | | | 150 | LLTLEEVTKDKPIR | Carbohydrate transport and metabolism |
| **3** | gi|4981998 | Oxidoreductase | | 45.0/20.0 | 5.77/4.55 | | 8 | 21% | | | 60 |  | Energy production and conversion |
| **4** | gi|4982186 | ATP synthase F0 | | 19.3/17.0 | 5.44/5.49 | | 14 | 45% | | | 98 | ALEQVQER  VFQDER | Energy production and conversion |
| **5** | gi|6919838 | 3-Oxoacyl-[acyl-carrier-protein] reductase | | 26.4/26.2 | 6.43/5.42 | | 6 | 29% | | | 58 |  | lipid metabolism |
| **6** | gi|4980562 | Oligopeptide ABC transporter, ATP-binding protein | | 37.5/19.3 | 7.66/4.56 | | 7 | 28% | | | 59 |  | Amino acid transport and metabolism |
| **Down-regulated** | | | |  | |  | | | |  | |  |  |
| **7** | gi|4980620 | Oxaloacetate decarboxylase | | 53.3/27.5 | 5.63/4.98 | | 10 | 22% | | | 59 |  | Carbohydrate transport and metabolism |
| **8** | gi|4981405 | Penicillin-binding protein 2 | | 65.0/66.0 | 6.07/5.32 | | 9 | 15% | | | 59 |  | Nucleotide transport and metabolism |
| **9** | gi|4981907 | Inosine-5'-monophosphate dehydrogenase | | 52.1/29.1 | 6.37/4.93 | | 10 | 33% | | | 66 |  | Nucleotide transport and metabolism |
| **10** | gi|9910756 | Adenylate kinase | | 25.4/25.0 | 6.24/4.91 | | 7 | 45% | | | 56 |  | Nucleotide transport and metabolism |
| **11** | gi|4981771 | Transcriptional regulator, LacI family | | 37.1/23.7 | 9.0/4.93 | | 8 | 23% | | | 58 |  | Transcription |
| **12** | gi|4981121 | Iron-dependent transcriptional repressor | | 17.8/14.2 | 8.73/4.79 | | 5 | 21% | | | 63 |  | Transcription |
| **13** | gi|4981294 | Transcriptional regulator, GntR family | | 14.1/31.2 | 8.66/4.86 | | 5 | 38% | | | 60 |  | Transcription |
| **14** | gi|4981613 | Transcriptional regulator, DeoR family | | 28.6/27.1 | 7.03/4.91 | | 5 | 18% | | | 65 |  | Transcription |
| **15** | gi|4981919 | Sensor histidine kinase | | 89.0/69.0 | 5.96/5.43 | | 12 | 16% | | | 62 | LFSESAGLALENAYNYENLR  QVLEFTEFNLNELIR  VILLSLVSER | Phosphorylation |
| **16** | gi|157879861 | Chain A, Solution Structure Of Tm1492, The L29 Ribosomal Protein | | 8.0/65.2 | 10.27/5.81 | | 3 | 40% | | | 60 |  | Translation, ribosomal structure and biogenesis |
| **17** | gi|7674078 | 33 KDa chaperonin | | 32.7/23.9 | 5.34/4.94 | | 8 | 23% | | | 55 |  | protein turnover, chaperones |
| **18** | gi|4981699 | Oligopeptide ABC transporter, ATP-binding protein | | 37.4/29.0 | 9.1/4.98 | | 5 | 15% | | | 60 |  | Amino acid transport and metabolism |
| **19** | gi|4981190 | Hypothetical protein | | 22.3/27.1 | 5.01/4.94 | | 8 | 53% | | | 88 |  | Unknown |
| **20** | gi|4981250 | Hypothetical protein | | 32.1/24.1 | 5.21/4.83 | | 6 | 33% | | | 59 |  | Unknown |
| **21** | gi|4982076 | Hypothetical protein | | 47.8/25.2 | 5.79/5.0 | | 5 | 17% | | | 54 |  | Unknown |
| **22** | gi|4982222 | Conserved hypothetical protein | | 22.7/23.7 | 6.62/4.99 | | 5 | 27% | | | 66 |  | Unknown |
| **Bell-shaped-regulated** | | |  | | |  | | | | | |  |  |
| **23** | gi|6226608 | Enolase | | 46.9/53.4 | 4.93/5.25 | | 9 | 24% | | | 92 |  | Carbohydrate transport and metabolism |
| **24** | gi|2687836 | F1F0-ATPase | | 51.2/54.1 | 4.92/4.76 | | 21 | 44% | | | 238 |  | Energy production and conversion |
| **25** | gi|14285794 | Probable peroxiredoxin | | 24.9/25.9 | 5.69/5.82 | | 8 | 33% | | | 77 |  | Energy production and conversion |
| **26** | gi|4982183 | ATP synthase F1 | | 31.9/29.5 | 7.74/6.21 | | 12 | 45% | | | 123 | FYEIPDFR | Energy production and conversion |
| **27** | gi|4981771 | Transcriptional regulator, LacI family | | 37.1/29.0 | 9.0/5.85 | | 5 | 15% | | | 60 |  | Transcription |
| **28** | gi|7388097 | DNA-directed RNA polymerase alpha chain | | 38.6/46.1 | 5.32/5.66 | | 9 | 23% | | | 109 |  | Transcription |
| **29** | gi|68565848 | Glutamine amidotransferase subunit pdxT | | 21.3/24.9 | 5.32/5.39 | | 9 | 51% | | | 128 |  | Amino acid transport and metabolism |
| **30** | gi|4981061 | Aspartokinase II | | 81.4/27.3 | 6.41/5.73 | | 9 | 15% | | | 52 |  | phosphorylation |
| **31** | gi|7388181 | 30S ribosomal protein S20 | | 11.3/32.2 | 10.65/4.89 | | 3 | 20% | | | 55 |  | Translation, ribosomal structure and biogenesis |
| **32** | gi|4981201 | Flagellar protein | | 18.7/18.1 | 5.39/5.13 | | 5 | 20% | | | 70 |  | Cell motility and secretion |
| **33** | gi|4981499 | LemA protein | | 21.6/28.8 | 6.02/5.83 | | 6 | 34% | | | 60 |  | Unknown |
| **34** | gi|4982178 | Conserved hypothetical protein | | 20.7/25.1 | 5.24/5.81 | | 7 | 40% | | | 59 | NVNTGEINLLYR  VIYDDDLVSFNVR | Unknown |
| **35** | gi|4981076 | Conserved hypothetical protein | | 20.8/22.5 | 5.34/5.30 | | 5 | 32% | | | 90 |  | Unknown |
| **36** | gi|4982272 | Conserved hypothetical protein | | 42.2/43.0 | 4.67/4.72 | | 6 | 22% | | | 58 | AEHIITLVLSGER | Unknown |
| **37** | gi|4982372 | Conserved hypothetical protein | | 32.8/35.1 | 5.2/5.48 | | 5 | 16% | | | 58 |  | Unknown |

Notes: The.:Theoretical value; Exp.:Experiment value.
